# Body composition of infants at 6 months of age using a 3-compartment model

**DOI:** 10.1038/s41430-023-01351-2

**Published:** 2023-10-13

**Authors:** Rebecca Kuriyan, Andrew P. Hills, Alexia Murphy-Alford, Ramya Padmanabha, Lukhanyo H. Nyati, Nuala M. Byrne, Anura V. Kurpad, Shane Norris, Multi-Center Infant Body Composition Reference Study (MIBCRS), Rebecca Kuriyan, Andrew P. Hills, Nuala M. Byrne, Anura V. Kurpad, Shane Norris, Shabina Ariff, Ina S. Santos, V. Pujitha Wickramasinghe, Alexia J. Murphy-Alford, Lukhanyo Nyati, Caroline S. Costa, Nishani Lucas, Tanvir Ahmad, Kiran D. K. Ahuja, Jeffrey M. Beckett, Renata M. Bielemann, Laila Charania, Michele P. Christian, Priscilla J. Divya, Anne Hanley, Manoja P. Herath, Leila C. Ismail, Sisitha Jayasinghe, Pulani Lanerolle, Cornelia Loechl, Najat Moktar, Upul Senerath, Christine Slater, Sajid Soofi, Steven J. Street, Neiva C. J. Valle, Ayesha Yameen

**Affiliations:** 1 St. John’s Research Institute, Bengaluru, India; 2 University of Tasmania, Hobart, TAS Australia; 3 International Atomic Energy Agency, Vienna, Austria; 4 University of Witwatersrand, Johannesburg, South Africa; 5 University of Southampton, Southampton, UK; 6 The Aga Khan University, Karachi, Pakistan; 7 Federal University of Pelotas, Pelotas, Brazil; 8 University of Colombo, Colombo, Sri Lanka; 9 Isotope Application Division, Pakistan, Institute of Nuclear Science and Technology (PINSTECH), Islamabad, Pakistan; 10 University of Sharjah, Sharjah, United Arab Emirates; 11 University of Oxford, Oxford, UK

**Keywords:** Nutrition, Paediatrics

## Abstract

**Background/Objectives:**

Two compartment (2 C) models of body composition, including Air Displacement Plethysmography (ADP) and Deuterium Dilution (DD), assume constant composition of fat-free mass (FFM), while 3-compartment (3 C) model overcomes some of these assumptions; studies are limited in infants. The objective of the present study is to compare 3 C estimates of body composition in 6-mo. old infants from Australia, India, and South Africa, including FFM density and hydration, compare with published literature and to evaluate agreement of body composition estimates from ADP and DD.

**Methods:**

Body volume and water were measured in 176 healthy infants using ADP and DD. 3C-model estimates of fat mass (FM), FFM and its composition were calculated, compared between countries (age and sex adjusted) and with published literature. Agreement between estimates from ADP and DD were compared by Bland–Altman and correlation analyses.

**Results:**

South African infants had significantly higher % FM (11.5%) and density of FFM compared to Australian infants. Australian infants had significantly higher % FFM (74.7 ± 4.4%) compared to South African infants (71.4 ± 5.0) and higher FFMI (12.7 ± 0.8 kg/m^2^) compared to South African (12.3 ± 1.2 kg/m^2^) and Indian infants (11.9 ± 1.0 kg/m^2^). FFM composition of present study differed significantly from literature. Pooled three country estimates of FM and FFM were comparable between ADP and DD; mean difference of −0.05 (95% CI: −0.64, +0.55) kg and +0.05 (95% CI: −0.55, +0.64) kg.

**Conclusions:**

3C-model estimates of body composition in infants differed between countries; future studies are needed to confirm these findings and investigate causes for the differences.

## Introduction

Body composition measurements in infants provide a critical baseline for understanding associations between growth in early life and the risk of disease throughout later life [1]. However, the accurate assessment of body composition in infancy is challenging, with errors due to unsuitable prediction equations, movement artefacts during imaging methods, and the unsuitability of many instruments for small infants. An optimal infant body composition measurement method needs to be practical, accurate, precise with minimal assumptions. Sexual dimorphism in relation to adiposity is established in early infancy and may be of relevance for later-life diseases [2, 3].

The 2C-model divides the body into FM and FFM, and 2 C techniques such as densitometry (body volume measurements by ADP) and isotope dilution (hydrometry, DD), have commonly been used to assess body composition in infants and children [4, 5]. While these techniques are practical and relatively simple, they assume values of FFM hydration or density, primarily based on studies conducted on infants from Western countries by Fomon et al. [6] and Butte et al. [7]; these assumed age- and sex-specific FFM density or hydration may not be appropriate universally, since many external factors can impact the process of growth and maturation [8, 9]. These measurements are limited in infants from diverse countries and ethnicities.

Multi-component models of body composition, combining measurements from different techniques, can be used to directly assess the FFM composition (water and mineral content) and thus explore the variations in FFM hydration and density. In the criterion four compartment (4 C) model [10, 11], total body water (TBW), bone mineral content (BMC) and body density (from body volume and weight), are assessed using hydrometry, dual-energy X-ray absorptiometry (DXA) and ADP, respectively. However, since DXA imaging involves exposure to a low dose of radiation and is affected by movement artefacts, it may not be appropriate for new-borns and young infants [2, 12]. On the other hand, the non-imaging 3-compartment (3 C) model [13], which combines measures of weight, body volume by ADP, and total body water by DD, avoids the assumption of water content of FFM between individuals and has been used safely in infants to yield estimates of FM, TBW and the anhydrous FFM (mineral, protein and DNA/glycogen) [14]. The 3 C model can be used in research settings to explore the assumed constants of density and hydration of FFM [6, 7] and provide important understanding and implications while applying 2 C models. Evaluation of the FFM density of Fomon et al. [6] against 3C-model estimates in a small number of Ethiopian infants aged 1.5–6 mo. [13], found them to be similar. However, to our knowledge, no study has simultaneously estimated body composition using the 3C-model in infants from countries with varied income and environmental settings, which could affect the FFM hydration or density.

This study firstly aimed to measure and compare the anthropometry and body composition of 6-mo. old infants from Australia, India and South Africa, using the 3C-model. Secondly, the study aimed to investigate the FFM density and hydration of infants and compare the values between countries and to published values [6, 7] Thirdly, the study aimed to evaluate the agreement of FM and FFM estimates between ADP and DD.

## Methods

This study included 6-mo. old infants from three countries, who were part of the larger multi-country Multi-center Infant Body Composition Reference Study (MIBCRS), which aimed to accurately assess body composition in healthy infants from birth to 2 y. The eligibility criteria for recruitment into MIBCRS were consistent with the WHO Multicentre Growth Reference Study (MGRS) [15], and full-term healthy infants (≥37 to ≤42 wk of gestation), weighing ≥2500 g, from singleton pregnancies of healthy non-smoker mothers planning to breastfeed for at least up to 6 mo., were recruited into the study. Infants with significant morbidity and congenital abnormalities were excluded. This multinational study fulfilled International Ethical Guidelines for Biomedical Research involving human participants, and each country site obtained approval from their local ethics review committee. Informed consent form was obtained from the mothers. The sample size of 44 infants in each country was estimated to observe a mean difference of 3.0% in %FM between different countries, with a SD of 3.9% and 4.2% [16], at 5% level of significance and 80% power, after correcting for the multiple comparison between 3 countries.

### Infant anthropometry

Infant weight on the day of measurement (at ~6 mo.) was measured using a pediatric electronic scale accurate to the nearest 0.01 kg (SECA 376, Hamburg, Germany, for Australian and South African infants; Salter 914, Delhi, India, for Indian infants). Length was measured to the nearest 0.1 cm using an infantometer (SECA 417, Hamburg, Germany). Standardized protocols were developed based on the WHO MGRS protocol [17], measurements were performed by staff who were initially trained at all sites by an international expert and then retrained every 3 months.

### Assessment of body density by ADP

Body volume, and thereby body density, was measured by ADP (PEA POD, Software version 3.5.0, 201, COSMED, USA), with a mean precision of 0.07%. Precision was measured at each study site using a standard 3 L volume phantom, provided by the manufacturer. The volume of the phantom was measured 10 times for three days, and the coefficient of variation (CV%) was calculated as standard deviation (SD)/ Mean. The mean CV% of the three countries was reported. Standard protocol was used at all sites [18]. The ratio of weight (kg) and the measured body volume (L) was used to calculate total body density (kg/L), from which the proportions of FM and FFM were calculated using assumed densities [6, 19] for each. FM and FFM were expressed as a percentage of body weight (% FM, % FFM), and in relation to height, as FMI (kg/m^2^) and FFMI (kg/m^2^).

### Assessment of TBW by DD

Infant TBW was determined by DD, where a dose of 1 g deuterium oxide (D_2_O; 99.8 atom %, Sigma-Aldrich, Canada) was orally administered to the infant using a syringe, following a baseline saliva sample, collected at least 15 min prior to their last feed. Saliva was sampled at 2.5 h and 3 h, allowing sufficient time for the isotope to equilibrate in the body water. The 3-h point was taken for the calculation [20]. Liquids were not provided to the infants between dosing and saliva sample collections. The collected saliva samples were stored in −20 °C freezer until analysis. The deuterium abundance in the thawed saliva samples was analyzed in duplicate by Fourier transformed infrared spectrometry (FTIR, Agilent 4500 Series, USA) in separate laboratories. Similar SOPs were followed in all countries and efforts were made to harmonize the equipment and protocol. Quality control of TBW analysis was assured by the regular site visits by IAEA technical expert and the interlab studies, which were comparable (Supplementary Table 1). TBW was calculated as the ratio of the administered dose of deuterium to its steady state enrichment in the body water, corrected for deuterium exchange into protein using a correction factor of 1.044 [21]. FFM was calculated from TBW assuming an age- and sex-specific hydration value of FFM [6, 22]. FM was calculated as the difference in body weight and FFM.

### Estimation of FM, FFM, density, and hydration of FFM, using the 3 C model

The 3C-model estimates of FM and FFM were derived using body weight (BW), body volume (BV, measured by ADP) and TBW (estimated from DD), assuming the density of fat (D_FM_) to be 0.9007 kg/L [19], and the density of water (D_W_) to be 0.9937 kg/L at body temperature [19]. The density (D_DB_) and composition of the ‘dry’ body (FM and the anhydrous FFM) were calculated using the Archimedes principle for binary mixtures with the proportions and assumed densities of fat (P_F_ and D_F_) and the density of anhydrous FFM (D_p+m_), rearranging to calculate the unknown fat proportion P_F_ as shown below.

$$\frac{1}{{D}_{{DB}}}=\frac{{P}_{F}}{{D}_{F}}+\frac{1-{P}_{F}}{{D}_{p+m}}$$

The density of the protein and mineral (D_p+m_) component was derived as 1.4959 kg/L, using sex-specific proportions and densities of protein (P_p_, D_p_) and mineral (P_m_, D_m_) in a 6-mo infant [19].

$$\frac{1}{{D}_{p+m}}=\frac{{P}_{p}}{{D}_{p}}+\frac{{P}_{m}}{{D}_{m}}$$

FFM density (DFFM) was calculated from the ratio of FFM and FFM volume as below:

$${{D}_{FFM}(kg/L)}=\frac{{Body\,weight\,(kg)}-{FM\,(kg)}}{{Body\,volume \,(L)}-{(FM/FM\,density)\,(L)}}$$

FFM hydration (HF) was calculated as ratio of measured TBW, and 3C-model estimate of FFM.

### Statistical analysis

Primary measurements were tested for normality assumptions using Q-Q plot and Shapiro Wilk test (*p* > 0.05). Continuous variables are presented as mean ± SD, while categorical variables are presented as percentage (%). Maternal and infant characteristics were compared between countries using Pearson’s Chi-square test and analysis of variance (ANOVA), as appropriate and multiple comparison was performed by 2 × 2 Chi-square (adjusted for *p* value) and Bonferroni correction method, respectively. The main effect of country and interaction effect of country x sex were analysed using Analysis of covariance (ANCOVA) to compare the infant anthropometry and body composition estimates from 3 C model, between the three countries adjusted for age and sex, followed by Bonferroni correction for multiple comparisons. The agreement of FM and FFM estimates obtained from ADP vs. DD were assessed using intraclass correlation (ICC) and the Bland–Altman method [23]. The one sample *t*-test was used to determine if the mean difference of FM estimates from ADP and DD were significantly different from zero. The estimated D_FFM_ and HF were compared with published data [6, 7] using the one sample *t*-test. The mean precision of the estimated FM (kg) using the 3 C model from the three countries, obtained from the propagation of error method was ±0.03 kg (1.3% of FM), calculated using the known error in measuring TBW from DD (0.02 kg), body weight (0.01 kg) and body volume (0.002 L). SPSS statistical package version 26.0 (SPSS Inc. Chicago, Illinois) was used. *P* < 0.05 was considered statistically significant.

## Results

The data were normally distributed; maternal and infant characteristics are presented in Table 1. Australian mothers were significantly older than mothers from India and South Africa. Majority (95%) of the Indian infants were exclusively breastfed (EBF) at 3 mo., while 61% and 21% of infants were EBF in Australia and South Africa, respectively. About 91% of Australian infants were Caucasian, while all infants from India were of Asian ethnicity and South African infants belonged to Black African ethnicity. There were no between-country body weight and length differences in infants at 6 mo. of age.

**Table 1:** Maternal and infant characteristics of the study population.

Variables	Australia (*n* = 46)	India (*n* = 86)	South Africa (*n* = 44)	Pooled (*n* = 176)	*P*-value
Maternal characteristics					
Maternal age (y)	30.3 ± 4.3^a,b^	25.8 ± 6.4^a^	25.1 ± 5.4^b^	26.8 ± 6.0*	<0.01
Gestational age (wk)	39.8 ± 1.0^a,b^	38.8 ± 0.9^a^	39.1 ± 1.4^b^	39.2 ± 1.2*	<0.01
Mode of delivery (%)					
Vaginal (spontaneous and assisted)	73.9	72.1	100.0	79.6	0.02
LSCS^d^	26.1	27.9	0.0	20.4	
Parity %					
Primiparous	35.2	57.0	56.1	53.4	0.17
Multiparous	64.8	43.0	43.9	46.6	
Infant characteristics					
Age (year)	0.49 ± 0.02^a,b^	0.51 ± 0.02^a,c^	0.50 ± 0.01^b,c^	0.50 ± 0.02*	<0.01
Weight at birth visit (kg)^e^	3.3 ± 0.4^a,b^	2.8 ± 0.3^a,c^	3.0 ± 0.3^b,c^	3.0 ± 0.4*	<0.01
Weight (kg)	7.3 ± 0.7	7.2 ± 0.8	7.2 ± 0.7	7.2 ± 0.8	0.75
Length (cm)	65.3 ± 2.2	66.0 ± 2.2	64.8 ± 2.9	65.5 ± 2.5	0.03
Sex %					
Female	41.3	51.2	65.9	52.3	0.06
Male	58.7	48.8	34.1	47.7	
Feeding practices at 3 mo. of infants age (%)					
Exclusively breastfed	60.9	94.9	21.4	66.9	<0.01
Exclusively formula fed	13.0	0.0	2.4	4.2	

Values are mean ± SD; *P* values using ANOVA, Pearson’s chi-square, or ANCOVA test as appropriate.

*P < 0.05;

^a^Within a row, superscripts denote significance between countries—Australia vs. India

^b^Within a row, superscripts denote significance between countries—Australia vs. South Africa

^c^Within a row, superscripts denote significance between countries—India vs. South Africa.

^d^Lower Segment Cesarean Section;

^e^0–8 d of postnatal age.

### 3C-model estimates of body composition

Body composition estimates obtained from the 3C-model for infants are presented in Table 2. Australian infants had significantly higher TBW (kg) and FFM (kg) when compared to Indian and South African infants, but %TBW adjusted for body weight (59.4 ± 4.1%), was only significantly higher than infants from South Africa (56.1 ± 4.6%). The %FFM of Australian infants (74.7 ± 4.4%) was significantly higher than infants from South African (71.4 ± 5.0%), while the %FM was significantly lower in Australian infants (25.3 ± 4.4%), when compared to South African (28.6 ± 5.0%). The FM (kg) was similar among the three countries. Infants from Australia had a significantly higher FFMI (12.7 ± 0.8 kg/m^2^) compared to infants from India (11.9 ± 1.0 kg/m^2^) and South Africa (12.3 ± 1.2 kg/m^2^). Pooled sex-specific estimates of the body composition showed that males had significantly higher TBW (kg), %TBW, FFM (kg), %FFM and FFMI, while females had significantly higher %FM. These results along with the within country sex-specific results are summarized in Supplementary Table 2.

**Table 2:** Body composition estimates using the 3C-model in infants from the three countries at 6 mo. of age.

	Australia (*n* = 46)	India (*n* = 86)	South Africa (*n* = 44)	Pooled (*n* = 176)	*P*-value
Primary parameters					
Weight (kg)	7.3 ± 0.7	7.2 ± 0.8	7.2 ± 0.7	7.2 ± 0.8	0.50
TBW (kg)	4.3 ± 0.4^a,b^	4.1 ± 0.5^a^	4.0 ± 0.4^b^	4.2 ± 0.4*	0.01
TBW %	59.4 ± 4.1^b^	57.7 ± 4.5	56.1 ± 4.6^b^	57.7 ± 4.5*	0.01
Body volume (kg/L)	7.2 ± 0.7	7.1 ± 0.8	7.1 ± 0.7	7.1 ± 0.8	0.63
Body composition estimates					
FM (kg)	1.9 ± 0.4	2.0 ± 0.5	2.1 ± 0.5	2.0 ± 0.5	0.13
FFM (kg)	5.4 ± 0.5^a,b^	5.2 ± 0.5^a^	5.1 ± 0.4^b^	5.2 ± 0.5*	0.01
% FM	25.3 ± 4.4^b^	27.4 ± 4.9	28.6 ± 5.0^b^	27.1 ± 4.9*	0.02
% FFM	74.7 ± 4.4^b^	72.6 ± 4.9	71.4 ± 5.0^b^	72.9 ± 4.9*	0.02
FMI (kg/m^2^)	4.3 ± 1.0	4.5 ± 1.1	5.0 ± 1.4	4.6 ± 1.2	0.07
FFMI (kg/m^2^)	12.7 ± 0.8^a,b^	11.9 ± 1.0^a^	12.3 ± 1.2^b^	12.2 ± 1.1*	<0.01
D_FFM_ (kg/L)	1.067 ± 0.006^b^	1.067 ± 0.006	1.071 ± 0.008^b^	1.068 ± 0.007*	0.03
HF	0.795 ± 0.015	0.795 ± 0.017	0.785 ± 0.020	0.792 ± 0.018	0.03

*D*_*FFM*_ Density of fat-free mass, *HF* Hydration factor, *FMI* Fat Mass Index, *FFMI* Fat-free mass index, *TBW* Total body water;

Values are mean ± SD; *P* values for main effects of country x sex using ANCOVA.

*P < 0.05,

^a^Within a row, superscripts denote significance between countries—Australia vs. India;

^b^Within a row, superscripts denote significance between countries—Australia vs. South Africa;

^c^Within a row, superscripts denote significance between countries—India vs. South Africa

### Density and hydration of FFM

The D_FFM_ of South African infants (1.071 ± 0.008 kg/L) was significantly higher than Australian infants (1.067 ± 0.006 kg/L, *P* value = 0.047), but not Indian infants (1.067 ± 0.006 kg/L, *P* = 0.087). The HF of South African infants (0.785 ± 0.020) was lower compared to Australian infants (0.795 ± 0.020), however, not significant. (*P* = 0.050). These results are reported in Table 2. Sex-specific analysis between countries (Supplementary Table 1) showed that South African females were significantly different from Indian females, but not Australian females in both D_FFM_ and HF.

When comparing the sex-specific D_FFM_ and HF estimates from the present study with those of Fomon et al. [6], only females from South Africa had significantly higher D_FFM_ and lower HF, while comparison of D_FFM_ and HF data from the present study with estimates from Butte et al. [7] showed that they were significantly different in infants from all three countries (Table 3).

**Table 3:** Sex-specific comparison of the density and hydration of fat-free mass (FFM) from the present study with published literature values [6, 7].

		Australia		India		South Africa		Pooled	
		Males *n* = 27	Females *n* = 19	Males *n* = 42	Females *n* = 44	Males *n* = 15	Females *n* = 29	Males *n* = 84	Females *n* = 92
Density of FFM (D_FFM_)kg/L	Present data	1.067 ± 0.006	1.067 ± 0.005	1.067 ± 0.006	1.068 ± 0.007	1.069 ± 0.008	1.072 ± 0.007	1.067 ± 0.007	1.069 ± 0.007
	Fomon et al. [6]	1.066	1.067	1.066	1.067	1.066	1.067	1.066	1.067
	*P* value	0.456	0.799	0.337	0.543	0.165	0.001*	0.073	0.009*
	Butte et al. [7]	1.062 ± 0.004	1.062 ± 0.006	1.062 ± 0.004	1.062 ± 0.006	1.062 ± 0.004	1.062 ± 0.006	1.062 ± 0.004	1.062 ± 0.006
	*P* value	0.001*	<0.001*	<0.001*	<0.001*	0.004*	<0.001*	<0.001*	<0.001*
Hydration of FFM (H_FFM_)	Present data	0.796 ± 0.017	0.795 ± 0.014	0.796 ± 0.016	0.794 ± 0.017	0.790 ± 0.021	0.783 ± 0.019	0.795 ± 0.017	0.790 ± 0.018
	Fomon et al. [6]	0.796	0.794	0.796	0.794	0.796	0.794	0.796	0.794
	*P* value	0.891	0.861	0.867	0.948	0.298	0.004*	0.460	0.058
	Butte et al. [7]	0.807 ± 0.012	0.807 ± 0.016	0.807 ± 0.012	0.807 ± 0.016	0.807 ± 0.012	0.807 ± 0.016	0.807 ± 0.012	0.807 ± 0.016
	*P* value	0.002*	0.001*	<0.001*	<0.001*	0.007*	<0.001*	<0.001*	<0.001*

Values are mean ± SD; One sample *t*-test;

*depicts statistical significance *P* < 0.05.

### Comparison of ADP and DD

**Fig. 1:**
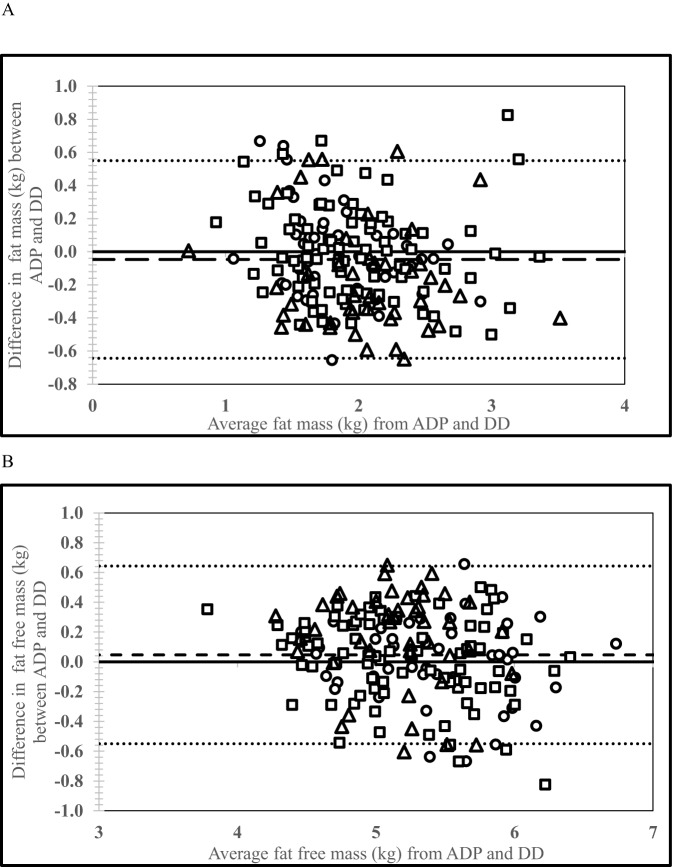
Bland–Altman plot comparing the body composition estimates of Air displacement plethysmography (ADP) vs. Deuterium dilution (DD) in infants from the three countries at 6 mo. of age. **A** Fat mass obtained from ADP vs. DD. **B** Fat-free mass obtained from ADP vs. DD. Circles denote Australia (*n* = 46), squares denote India (*n* = 86) and triangles denote South Africa (*n* = 44). The black solid line refers to zero. The black dashed line represents the mean difference (−0.05 kg) for FM and (+0.05 kg) for FFM, which was significantly different (*P* < 0.05), from zero. The limits of agreement are represented by dotted lines representing upper and lower limits of 95% CI: −0.64, +0.55 kg for FM and −0.55, +0.64 kg for FFM.

The ICC of pooled FM and FFM estimates obtained from ADP and DD was high (0.90, 95% CI: 0.86–0.92 and 0.91, 95% CI: 0.88–0.94, respectively). The mean difference in the pooled FM and FFM estimates obtained from DD and ADP was −0.05 ± 0.30 (95% CI: −0.64, +0.55) kg and +0.05 ± 0.30 (95% CI: −0.55, +0.64) kg respectively, which were significantly different from zero (*P* < 0.05) (Fig. 1). The mean difference between the two methods for Australia, India, and South Africa were 0.00 ± 0.27 (95% CI: −0.55, +0.54) kg, −0.01 ± 0.28 (95% CI: −0.57, +0.55) kg, −0.17 ± 0.33 (95% CI: −0.82, +0.49) kg, respectively for FM and 0.00 ± 0.27 (95% CI: −0.54, +0.55) kg, +0.01 ± 0.28 (95% CI: −0.55, +0.57) kg, 0.17 ± 0.33 (95% CI: −0.49, +0.82) kg, respectively for FFM. The correlation between the difference and average of FM derived from ADP and DD was inverse and significant (*r* = −0.18, *p* = 0.02; *y* = −0.1108x + 0.171), while it was linear and significant for FFM (*r* = +0.18, *p* = 0.02; *y* = −0.1059x + 0.6034).

## Discussion

This paper presents, for the first time, 3C-model body composition estimates of 6 mo. infants recruited from three different countries in a simultaneous multicenter study. The rank order of FFMI was Australia > South Africa > India, while for FMI, it was South Africa > India > Australia. Infants from South Africa had significantly higher density of FFM when compared to infants from Australia. Sexual dimorphism existed in South African infants, females had a significantly higher D_FFM_ and lower HF when compared to published estimates [6]. Pooled FM (kg) and FFM (kg) estimates from the three countries, measured by ADP and DD, had good reliability and relatively low differences.

Measurement of body composition at 6 mo. can provide insights on the effect of feeding patterns on body composition. A systematic review summarized the effect of infant feeding (breast feeding vs formula feeding) on infant (0–12 mo.) body composition and observed that formula-fed infants had lower FM when compared to breast fed infants at 3–4 mo. and 6 mo., but there was a trend toward higher FM in formula-fed infants at 12 mo. of age [24]. Similar results were observed in a large, mother-baby dyad group in USA, where breastfed babies had higher percent body fat at 6 mo. of age [25]. Breast fed infants were observed to have higher circulating leptin in comparison to formula-fed infants at ages <4 mo., but not in later infancy [26, 27], which could be a reason for the observed higher FM in breast fed infants during early infancy. Similarly, breastfeeding was associated with lower FFM in infants between 3–6 mo. [24, 28] and at 6 mo., when compared to formula feeding [24]. The higher protein content of formula feeds compared to breast milk [25] may explain the higher accrual of FFM in early life, however, these findings need to be prospectively confirmed in longitudinal studies.

In the present study, sex-based differences in D_FFM_ and HF were only observed in South African infants, however, since males only comprised 34% (*n* = 15), results need to be considered with caution. Earlier studies [7, 29, 30] have not observed any significant sex-specific differences and it has been suggested that attempts to identify sex-based differences in the composition of FFM in infants may not be desirable unless the methodology was perfect [31].

Most body composition reference data for infants have been published using [13, 18, 32–34] the 2 C model, which assumes constant values for the composition of FFM [6, 7]. The values from Fomon et al. [6], were based on data from different published reports, modeled with many assumptions to derive reference data for D_FFM_ and HF. Butte et al. values [7] were based on longitudinal data from infants of US aged birth to 2 y. The D_FFM_ of infants from all three countries in the present study were significantly higher, while HF estimates were significantly lower than those from Butte et al. [7]. Similarly, lower body FM estimates at 12 wk of age [35], was observed when using the D_FFM_ value from Butte et al. [7] in comparison to Fomon et al. [6], being particularly evident at 1 wk of age, which could have implications for estimates of FM and FM accretion in early life. However, the biological importance of these findings may be more relevant rather than just the statistical significance and hence population specific D_FFM_ and HF, which may be best applicable for each country should be used.

Estimates of body composition measured by ADP and DD in the present study showed good reliability, with a low mean difference, concurring to results observed in infants aged 0.5 to 6 mo., where no significant differences were observed between percent FM estimates obtained from both methods [36]. While DD is accurate for estimating body composition in infancy, it is not practical in infants under 3 mo. due to difficulties in dosing or collecting saliva samples. ADP is a safe and rapid measurement, validated using the 4C-model in term infants [37, 38], but being practical only for infants weighing up to 8 kg. The results from the two methods should, however, not be used interchangeably while measuring body composition in infants, and may be reliable only for population estimations rather than individual measurements.

Limitations of the present study were the single DD measurement and the lack of feeding data at 6 mo. Factors such as socio-economic status, feeding patterns, ethnicity, and climate, which may have contributed, were not considered in the analysis. In conclusion, accurate estimates of body composition in infants from three countries, using 3 C model suggests some significant between-country variability, however, in view of the significant demographic, social, and environmental differences among the countries, larger studies are needed to investigate the true causes of the differences. Estimates using ADP and DD correlated well with relatively low bias, however, the wide 95% CI suggests that these techniques may be more reliable for population estimates. The D_FFM_ of South African infants differed from Australian infants and from estimates from Fomon et al. [6], while all countries differed from estimates of Butte et al. [7]. This highlights the need for further investigation of the assumed FFM variables in infancy, which may have important implications for the different 2 C models used in infant research.

## Supplementary information

Supplementary Table 1

Supplementary Table 2

## Data Availability

Data described in the manuscript, code book, and analytic code will be made publicly and freely available without restriction at, https://osf.io/r5pbn, OSF | Body composition of infants at 6 months of age using a 3-compartment model.
