# Supplementary material for: Body composition of infants at 6 months of age using a 3-compartment model
Source: Eur J Clin Nutr. 2023 Oct 13;78(11):936–42. doi: 10.1038/s41430-023-01351-2 (PMC11537952; doi:10.1038/s41430-023-01351-2)
Supplement: Supplementary file 1 — Supplementary Table 1 [file 41430_2023_1351_MOESM1_ESM.docx]

**Supplementary Table 1: Inter-laboratory Z-scores of deuterium enrichment.**

|  | **Sample 1** | **Sample 2** | **Sample 3** | **Sample 4** |
| --- | --- | --- | --- | --- |
| **Global Mean ± SD (ppm)** | **309.3 ± 17.63** | **981.3 ± 50.60** | **113.9± 12.24** | **308.4 ± 17.56** |
| **Australia** | 0.71 | 0.70 | 0.39 | 0.77 |
| **India** | -0.47 | -0.45 | -0.39 | -0.39 |
| **South Africa** | 0.90 | 1.03 | 0.32 | 0.93 |

The Z score of each sample was computed in comparison to the deuterium enrichment of the global mean (Z score=0)
