# Supplementary material for: Body composition of infants at 6 months of age using a 3-compartment model
Source: Eur J Clin Nutr. 2023 Oct 13;78(11):936–42. doi: 10.1038/s41430-023-01351-2 (PMC11537952; doi:10.1038/s41430-023-01351-2)
Supplement: Supplementary file 2 — Supplementary Table 2 [file 41430_2023_1351_MOESM2_ESM.docx]

# Supplementary Table 2: Sex-specific between and within country comparison of anthropometric and body composition estimates using the 3C model in infants from the three countries at 6 mo. of age

|  | **Australia** | | **India** | | **South Africa** | | **Pooled** | |
| --- | --- | --- | --- | --- | --- | --- | --- | --- |
|  | **Females**  *n*=19 | **Males**  *n*=27 | **Females**  *n*=44 | **Males**  *n*=42 | **Females**  *n*=29 | **Males**  *n*=15 | **Females**  *n*=92 | **Males**  *n*=84 |
| **Weight (kg)** | 7.0 ± 0.5 | 7.5 ± 0.7^#^ | 6.8 ± 0.7 | 7.5 ± 0.8^#^ | 7.1 ± 0.6 | 7.4 ± 0.8 | 7.0 ± 0.7 | 7.5 ± 0.8^#^ |
| **Length (cm)** | 64.1 ± 2.0 | 66.2 ± 2.0^#^ | 65.3 ± 2.2^c^ | 66.8 ± 2.1^#^ | 63.9 ± 2.6^c^ | 66.6 ± 2.7^#^ | 64.6 ± 2.4^*^ | 66.6 ± 2.2^#^ |
| **TBW (kg)** | 4.1 ± 0.3 | 4.5 ± 0.4^#^ | 3.9 ± 0.4 | 4.4 ± 0.4^#^ | 3.9 ± 0.3 | 4.2 ± 0.4^#^ | 3.9 ± 0.4 | 4.4 ± 0.4^#^ |
| **TBW %** | 58.7 ± 4.0^b^ | 60.0 ± 4.1 | 57.1 ± 4.6 | 58.4 ± 4.3 | 55.0± 4.2 ^b^ | 57.6 ± 4.9 | 56.9 ± 4.5^*^ | 58.8 ± 4.4^#^ |
| **FM (kg)** | 1.8 ± 0.4 | 1.9 ± 0.4 | 1.9 ± 0.5 | 2.0 ± 0.5 | 2.1 ± 0.5 | 2.0 ± 0.6 | 2.0 ± 0.5 | 2.0 ± 0.5 |
| **FFM (kg)** | 5.1 ± 0.4 | 5.7 ± 0.5^#^ | 4.9 ± 0.5 | 5.5 ± 0.4^#^ | 5.0 ± 0.3 | 5.3 ± 0.4^#^ | 5.0 ± 0.4 | 5.5 ± 0.5^#^ |
| **% FM** | 26.2 ± 4.5 | 24.6 ± 4.2 | 28.1 ± 5.0 | 26.7 ± 4.9 | 29.3 ± 5.0 | 27.2 ± 5.0 | 28.1 ± 5.0 | 26.1 ± 4.7^#^ |
| **% FFM** | 73.8 ± 4.5 | 75.4 ± 4.2 | 71.9 ± 5.0 | 73.3 ± 4.9 | 70.7 ± 5.0 | 72.8 ± 5.0 | 71.9 ± 5.0 | 73.9 ± 4.7^#^ |
| **FMI (kg/m^2^)** | 4.5 ± 1.0 | 4.2 ± 0.9 | 4.5 ± 1.1^c^ | 4.5 ± 1.1 | 5.2 ± 1.3 ^c^ | 4.6 ± 1.4 | 4.7 ± 1.2* | 4.5 ± 1.1 |
| **FFMI (kg/m^2^)** | 12.5 ± 0.5 ^a^ | 12.9 ± 1.0 ^b^ | 11.5 ± 0.8 ^ac^ | 12.3 ± 0.9^#^ | 12.3 ± 1.3 ^c^ | 12.1 ± 1.1 ^b^ | 12.0 ± 1.0^#*^ | 12.5 ± 1.0^#*^ |
| **D_FFM_ (kg/L)** | 1.067 ± 0.005 | 1.067 ± 0.006 | 1.068 ± 0.007 ^c^ | 1.067 ± 0.006 | 1.072 ± 0.007 ^c^ | 1.069 ± 0.008 | 1.069 ± 0.007* | 1.067 ± 0.007 |
| **HF** | 0.795 ± 0.014 | 0.796 ± 0.017 | 0.794 ± 0.017 ^c^ | 0.796 ± 0.016 | 0.783 ± 0.019 ^c^ | 0.790 ± 0.021 | 0.790 ± 0.018* | 0.795 ± 0.017 |

^1^Values are mean ± SD ; Independent sample t-test; ^#^  different from female,  *P*<0.05;

^2^One -way ANOVA ^*^ *P*<0.05 denotes between-country sex-specific comparison. Within a row, superscripts denote significance between countries - ^a^ Australia vs. India, ^b^ Australia vs. South Africa; ^c^ India vs. South Africa.
